# Progress in Surgery

**Published:** 1896-02-08

**Authors:** 


### Progress in Surgery.

### BRAIN SURGERY (*Continued*).

**Operations for the Relief of Intra-Cranial Tension.**—Much has recently been written in recommendation of operative interference in conditions of increased intra-cranial tension from one cause or another. Mr. Parkin,[1] of Hull, has added two more cases to the list of operations performed for hydrocephalus. The operation, he says, is simple, quickly performed, and involves no serious risk in itself. The results also are immediate. The drainage should be gradual and continued for some days. In the case of a boy, three and a half years old, who suffered from chronic hydrocephalus, there were frequent fits of a general character deficient intelligence, inability to talk or walk, and want of control of the organic reflexes. A hole, the size of a sixpenny piece, was made on one side of the median line of the occipital bone, the dura opened, a probe passed into the fourth ventricle, to ensure patency of the foramen of Majendie, and a small silk drain passed under the cerebellum. The drain was kept in for twenty-eight days. Ten months after "he could talk fairly well, recognise different objects and persons, and could walk if assisted with the hand. Since the operation he has grown two inches and a half, and the bodily increase in size, along with the absence of cranial disease, has almost removed the disparity which previously existed."

The other case, a boy five months old, was less satisfactory. There was evidence of congenital syphilis, and the patient had fits and enlargement of the head. Drainage was established, and for a few days improvement took place, but vomiting and hyperpyrexia ensued six days after the operation from too copious drainage, and the child died.

A very similar case to the former of these was reported in great detail by Glynn and Thomas, of Liverpool,[2] a week earlier. A young man of eighteen had for a year and a half suffered from occasional attacks of severe headache and giddiness, the severity and frequency of the attacks having greatly increased within the last six months, until he had constant pain, was unable to stand, and his memory, sight, and hearing had gradually failed. The clinical symptoms indicated a lesion in the right cerebellar fossa, and accordingly an opening was made through the occipital bone. The operation was complicated by great hæmorrhage and respiratory troubles. It was completed by opening the dura and passing the finger in the direction of the fourth ventricle, when a soft bulging was felt. This gave way and a considerable quantity cf fluid escaped. Immediately the respiration became normal and the bleeding became less profuse. The assumption was that the posterior medullary vellum had been opened through, and an internal hydrocephalus relieved by restoring the communication with the fourth ventricle. On regaining consciousness the headache had gone and the other symptoms gradually passed off.

A case of hydrocephalus associated with cerebral tumour is recorded by Dinkler.[3] A boy of four was found to have distinct weakness in the upper part of the body and head, the weakness being relieved by sitting, standing, or walking. He also lost control of bladder and rectum. He had a fairly big head, sluggish pupils, swollen discs, weakness of right side of face and right limbs, with increased knee jerk on the right side. Following this there were sick attacks, with loss of consciousness, and later epileptiform attacks. The intra-cranial tension was relieved by puncture between the third and fourth lumbar vertebræ, but the child died unrelieved soon after. The diagnosis had been that of cerebellar tumour with hydrocephalus, but at the post-mortem examination a large tumour of the right cerebral hemisphere, with severe internal hydrocephalus, was revealed. The left cortex was degenerated, and the right parietal cortex atrophied by pressure. The posterior roots and column were also degenerated.

Angellucci[4] reports three cases of blindness due to œdema of the optic disc in which trephining was followed by great improvement in vision. Two of the cases were cerebellar tumours.

**Tubercular Meningitis.**—Parkin[1] briefly refers to four cases of tubercular meningitis which he has treated by tapping. All the patients were comatose when operated on, but regained consciousness, were free from pain, and lived for a longer time than was expected otherwise. Gautley[5] has operated unsuccessfully on a case. He says: If operative interference is of value, it is only in cases characterised by copious effusion. Large effusions are more common in cases of acute simple meningitis than in those due to tubercular infection. Operative interference should only be undertaken when there is evidence of intra-cranial pressure, and it is advisable to operate before the supervention of coma, during the stage of irritation. Tubercular meningitis is almost always part of an acute general tubercular infection, generally secondary to breaking-down caseous glands at the root of the lungs or in the mesentery, and operative treatment in such cases is unlikely to result in more than temporary amelioration of the symptoms. The operation does produce temporary improvement, and is more likely to result in a successful issue than the expectant treatment generally adopted.

Angel[6] advocates craniotomy in cases of profound loss of consciousness, due to such conditions as intra-cranial hæmorrhage, hæmatoma of dura mater, &c.

**Craniectomy.**—Barbour[7] contributes a paper, in which he discusses the present position of craniectomy in the treatment of idiocy. The operation has for its object the removal of more or less of the vault of the skull to permit of expansion of the brain, and is based on the assumption that the cerebral deficiency is secondary to early synostosis of the cranium. The writer sums up as follows: (1) Premature synostosis of the cranium does not occur in idiocy; (2) the arrested growth of the skull is due to the arrested development of the brain, and not *vice versâ*; (3) the lesions to which idiocy are due are profound, varied, extensive, and by no means susceptible of relief by craniectomy; (4) the results obtained by operation are slight, doubtful, or nil. Over against this operation he would write, "Mene, mene, tekel upharsin." Spanbock[8] records a case of imbecility improved by craniectomy. The improvement began after two months, and was both intellectual and moral, the patient's previous tendency to stealing, lying, and abusive language has disappeared, and he is now industrious, trustworthy, and grateful. Dr. Telford Smith[9] also sums up against the operation of craniectomy, although he thinks the experiment was justifiable. Dr. Shuttleworth thinks there is no *rationale* for such an operation in the majority of cases of microcephalus, which, as a rule, depend on prenatal defect of brain development. Cases of premature synostosis causing microcephalus are very exceptional. Special training is of much more service in promoting mental improvement.

**Cerebral Tumours.**—Successful operations for cerebral tumours have recently been recorded by Kappelen,[10] who removed an endothelioma from the fissure of Rolando, which had given rise to Jacksonian spasms; Dana and Conway[11]; and Syme,[12] an Australian surgeon, who removed a tumour from the dura mater which by pressure had given rise to sensory and motor symptoms referable to the face, tongue, and throat centres. Gajkiewick[13] removed a gummatous growth from the region of the face and arm centre. Anti-syphilitic remedies had only produced a slight and transient benefit, and as the Jacksonian epileptic fits, which always began in the upper extremity of the left side, returned with increased severity, and optic neuritis developed the operation was undertaken. After the operation anti-syphilitic remedies were still given to check slight symptoms, but the patient completely recovered.

[1] Lancet, Nov. 9, 1895. [2] Lancet, Nov. 2, 1895. [3] Deutsche Zeitschrift f. Nervenhulkunde. [4] Archiv. di Oftalmol., 12, 1995. [5] Med. Week, 1895, p. 362. [6] Ep. B.M.J., July. 1895. [7] Amer. Med. Surg. Bullet., Oct., 1895. [8] Ep. B.M.J., Nov. 2, 1895. [9] B.M.J., Sep., 1895. [10] Central-Blatt f. Chirurg., No. 38, 1895. [11] N.Y. Med. Jour., June, 1895. [12] Amer. Jour. Med. Sciences, Sep., 1895. [13] Lancet, Oct. 5, 1895.

